# Comparison of Pregnancy and Neonatal Outcomes of Single Frozen Blastocyst Transfer Between Letrozole-Induction and HRT Cycles in Patients With Abnormal Ovulation

**DOI:** 10.3389/fendo.2021.664072

**Published:** 2021-04-16

**Authors:** Junwei Zhang, Zhen Li, Lijun Sun, Yichun Guan, Mingze Du

**Affiliations:** The Reproduction Center, The Third Affiliated Hospital of Zhengzhou University, Zhengzhou, China

**Keywords:** letrozole, frozen embryo transfer, live birth rate, miscarriage, neonatal outcomes

## Abstract

**Background:**

The use of frozen embryo transfer (FET) cycles has dramatically risen. The optimal endometrial preparation method for women undergoing FET is of utmost importance to provide the optimal chances of pregnancy. For patients with abnormal ovulation in particular, there have been few studies on FET protocols; notably, most of these studies focus only on the clinical pregnancy rate or live birth rate (LBR) and pay little attention to the regimen’s safety for offspring.

**Methods:**

It was a retrospective cohort study. First FET cycle with a single blastocyst from whole embryo frozen IVF/ICSI at the Reproductive Center of Third Affiliated Hospital of Zhengzhou University between January 2016 and January 2020. The LBR was the primary outcome of interest. The secondary outcome measures were miscarriage rate and offspring safety, including preterm birth, low birthweight (LBW), small-for-gestational age (SGA), macrosomia and large-for-gestational age (LGA).

**Results:**

In total, 2782 FET cycles met the eligibility criteria for analysis. Additionally, there were 1178 singleton births from FET cycles. The clinical pregnancy rate was 58.4% in the L-FET group and 54.5% in the HRT group, with no statistical significance (P=.116). The miscarriage rate was higher in the HRT group than in the L-FET group (21.7% vs. 14.3%, P=.005). The LBR was significantly higher in the L-FET group than in the HRT group (49.6% vs. 41.7%, P=.001). Neonatal outcomes were similar between the two groups. After adjustments for confounding factors, the LBR was higher in the L-FET group (aOR 1.30, 95% CI 1.06-1.58). The rate of miscarriage was lower in the L-FET group (aOR 0.63, 95% CI 0.44-0.90).

**Conclusion:**

For patients with abnormal ovulation, the L-FET regimen has a higher LBR and lower miscarriage rate than HRT. The neonatal outcomes were similar between the two groups.

## Introduction

Over the past decade, the use of frozen embryo transfer (FET) cycles has dramatically risen with the development of vitrification technology, and a rapid rise in single embryo transfer (ET), combined with the development of preimplantation genetic testing(PGT), has increased the number of embryos available for freezing (1–3). FET increases the cumulative live birth rate (LBR), reduces cost, is relatively simple to undertake and can be accomplished in a shorter time period than repeated *in vitro* fertilization (IVF) or intracytoplasmic sperm injection (ICSI) cycles with fresh ET. Currently, for every 2 embryos transferred, one is a FET (4). In some countries, the number of FET cycles far exceeds the number of fresh ET cycles (5).

The optimal endometrial preparation method for women undergoing FET is of utmost importance to provide the optimal chances of pregnancy. There are different FET cycle regimens used to prepare the endometrium, including natural cycles, hormone replacement therapy (HRT) with estrogen and progesterone, cycles in which ovulation is induced by drugs, and gonadotrophin-releasing hormone agonist (GnRH-a) cycles. Although there are many options for the preparation of the endometrium, there is still no uniform recommendation for the preparation of the FET endometrium for different groups of women to date (6–8). For patients with abnormal ovulation in particular, there have been few studies on FET protocols; notably, most of these studies focus only on the clinical pregnancy rate or LBR (9–11) and pay little attention to the regimen’s safety for offspring. The most frequently used regimens for patients with abnormal ovulation are ovulation induction by letrozole and HRT.

Letrozole is a third-generation aromatase inhibitor that is used mainly for the treatment of postmenopausal breast cancer. Recent studies have shown that letrozole can be used effectively for ovulation induction. Letrozole has a short half-life and has both peripheral and central effects (12, 13). Similar to clomiphene, Letrozole has no antiestrogen effect, is effective at promoting ovulation and has little effect on the cervical mucus, endometrium and sex hormone levels (13, 14). Furthermore, there are no obvious teratogenic effects on the fetus (15, 16). HRT can be used to prepare the endometrium, and then, the date of transplantation can be arranged and the number of monitoring procedures reduced; additionally, this is a popular clinical method for people with ovulation disorders (4).

However, there are few studies comparing the two regimens in women with ovulation disorders, and the existing ones focus mostly on clinical outcomes, such as the clinical pregnancy rate or LBR (9, 17). Moreover, few studies have focused on or compared the safety of the two regimens for offspring. To the best of our knowledge, only one study has investigated the safety of the two regimens for offspring, but the study did not implement restrictions of the inclusion of patients; that is, patients with either normal or abnormal ovulation were included. Additionally, the study was limited by its lack of important information, including the dose and duration of letrozole (18). Therefore, the purpose of this study was to explore the clinical outcomes and safety to offspring of L-FET and HRT for patients with ovulation disorders and provide evidence to guide the choice of clinical protocol.

## Materials and Methods

### Study Design and Population

This was a retrospective cohort study approved by the review board of the Third Affiliated Hospital of Zhengzhou University. All patients who initiated the first FET of whole-embryo-cycle transfer with first IVF/ICSI cycle at the Reproductive Center of Third Affiliated Hospital of Zhengzhou University between January 2016 and January 2020 were analyzed for potential inclusion. We included FET cycles of oligo-anovulation or anovulation with L-FET or HRT, and only single blastocyst transfer cycles were included. Cycles with maternal age >40 years were excluded, and cases with adenomyosis, uterine malformations, endometrial polyps, and PGT were excluded.

### Endometrial Preparation Protocols

Beginning on menstrual cycle day 3, 4 or 5, 2.5 mg of letrozole (Hengrui Medicine Co., China) was administered daily for 5 consecutive days. Then, follicle growth was monitored by vaginal ultrasound and, if necessary, combined with serum estradiol analysis on day 10. If the diameter of the dominant follicle was greater than 14 mm, there was no need to add HMG to the regimen; otherwise, HMG (Lizhu Pharmaceutical Trading Co., China) 37.5-75 IU daily was added according to the development of the follicle. When the diameter of the dominant follicle was greater than 18 mm, the endometrial thickness was greater than 7 mm, serum estradiol was >150 pg/ml and the occurrence of an LH surge was evident, 10 000 IU urinary hCG was injected (Lizhu Pharmaceutical Trading Co., China). FET was cancelled for cycles with insufficient thickness (<7 mm) or insufficient follicle development (1. The follicle was ≤14mm; 2. the estrogen was ≤150pg/ml; 3. The follicle was atrophy.).

For HRT cycles, vaginal ultrasound examination was performed on the 3rd day of the menstrual cycle, 2-3 mg of estradiol valerate was taken orally three times daily (Bayer Co. Germany), and vaginal ultrasound examination was performed 7 days later. The drug dose was adjusted according to the thickness of the endometrium (up to 9 mg per day). When the medication was taken for > 12 days, the endometrial thickness was ≥7 mm, and the serum estrogen level was greater than 150 pg/ml, endometrial transformation was performed. FET was cancelled for Cycles with insufficient thickness (<7 mm).

After the hCG injection in the L-FET cycle or after a medication time of > 12 days in the HRT cycle, an endometrial thickness of ≥7 mm was verified, and routine corpus luteum support, namely oral dydrogesterone (2 times daily, 10 mg once) (Abbott Co. America) and intravaginal administration of 90 mg of a progesterone sustained-release vaginal gel (Merck Co. Germany), was given. Five days after endometrial development with corpus luteum support, single blastocyst transplantation was carried out by abdominal ultrasound. Corpus luteum support was performed at least until 55 days after transplantation if pregnancy occurred.

### Outcome Measures and Definition

The primary outcome measure was LBR, defined as at least one live birth after ≥ 28 gestational weeks.

The secondary outcome measures were the miscarriage rate (defined as a loss of clinical pregnancy before 28 gestational weeks) and offspring safety, which was assessed by examining the neonatal birth weight of singleton live births as follows: low birthweight (LBW, birthweight<2500 g), small-for-gestational age (SGA, <10th percentile for gestational age) (19), macrosomia (birthweight≥4000 g), and large-for-gestational age (LGA, >90th percentile for gestational age) (19).

### Statistical Analysis

All statistical management and analyses were performed using SPSS software, version 22.0.

The one-sample K-S test was used to check for normality. Continuous variables with abnormal distributions are expressed as the mean ± SD, and Student’s t test was used to assess between-group differences. Categorical variables are represented as the number of cases (n) and percentage (%). The means from chi-square analyses were used to assess the differences between groups with Fisher’s exact test when necessary. For outcome measures (LBR, miscarriage rate, preterm birth, LBW, SGA, macrosomia, LGA), multiple logistic regression was used to adjust for the baseline characteristics. Unadjusted odds ratios and adjusted odds ratios (AORs) with 95% confidence intervals (CIs) were calculated. Statistical significance was set at p<0.05.

## Results

### Study Population

From January 2016 to January 2020, a total of 2782 FET cycles, including 502 L-FET and 2280 HRT cycles, met the eligibility criteria for analysis. There were 1178 FET cycles with singleton births, including 245 cycles from L-FET cycles and 933 from HRT cycles.

### Baseline Characteristics

**Table 1** lists the cycle baseline characteristics. There were no significant between-group differences in maternal age, paternal age, body mass index, duration of infertility, type of infertility, infertility diagnosis, basal serum FSH level, basal antral follicle count, fertilization method or developmental stage of the blastocyst. However, the endometrial thickness on the day of embryo transfer in the L-FET group was significantly higher than that in the HRT group (P<.001).

**Table 1:** Basic characteristics for cycles from 2017 through 2019.

Characteristics	L-FET (n=502)	HRT (n=2280)	*P* value
Maternal age(y)	30.95±4.56	31.20±4.97	.062
Paternal age(y)	31.67±5.14	32.33±5.72	.058
Body mass index(kg/m2)	23.88±2.99	23.97±3.18	.548
Duration of Infertility (y)	3.26±2.83	3.39±2.75	.374
Type of infertility			.239
Primary infertility	46.6(234/502)	43.7(997/2280)	
Secondary infertility	53.4(268/502)	56.3(1283/2280)	
Infertility diagnosis			.322
Tubal factor	32.9(165/502)	35.4(807/2280)	
Male factor	21.7(109/502)	19.0(434/2280)	
Others	45.4(228/502)	45.6(1039/2280)	
Basal serum FSH level(IU/L)	5.5±1.7	5.8±2.1	.077
Basal antral follicle count	19.97±7.03	18.90±7.52	.072
Fertilization method			.609
IVF	73.5(369/502)	74.6(1701/2280)	
ICSI	26.5(133/502)	25.4(579/2280)	
Endometrial thickness on day of embryo transfer (mm)	9.72±1.83	8.99±2.10	<.001
Development stage of blastocysts			.323
D5	63.9(321/502)	61.1(1404/2280)	
D6	36.1(181/502)	38.4(876/2280)	

Data are presented as mean ± SD for continuous variable and % (n/N) for categorical variable. Student t test was used for continuous variables, and the Pearsonχ^2^ test was used for categorical variables with Fisher’s exact test when necessary.

### Reproductive Outcomes

As shown in **Table 2**, the clinical pregnancy rate was 58.4% for the L-FET group and 54.5% for the HRT group, with no statistical significance (P=.116). The miscarriage rate was higher in the HRT group than in the L-FET group (21.7% vs. 14.3%, P=.005). Additionally, the LBR was significantly higher in the L-FET group than in the HRT group (49.6% vs. 41.7%, P=.001); similarly, the singleton LBR was higher in the L-FET group (48.8% vs. 40.9%, P=.001). The twin pregnancy rates were comparable between the two groups (0.8% vs. 0.8%, P=.987). Neonatal outcomes, including the newborn sex ratio and rates of preterm birth, normal neonatal birthweight, LBW, SGA, macrosomia and LGA, were similar between groups. The specific values are presented in **Table 3**.

**Table 2:** Comparison of pregnancy outcome between the two groups.

	L-FET (n=502)	HRT (n=2280)	*P* value
Clinical pregnancy rate	58.4(293/502)	54.5(1243/2280)	.116
Abortion rate	14.3(42/293)	21.7(270/1243)	.005
Live birth rate	49.6(249/502)	41.7(951/2280)	.001
Singletons	48.8(245/502)	40.9(933/2280)	.001
Twins	0.8(4/502)	0.8(18/2280)	.987

Data are presented as % (n/N) for categorical variable. Pearsonχ^2^ test was used for categorical variables with Fisher’s exact test when necessary.

**Table 3:** Comparison of singleton neonatal outcome between the two groups.

	L-FET (n=245)	HRT (n=933)	*P* value
Gender of newborn			.570
Male	53.1(130/245)	55.1(514/933)	
Female	46.9(115/245)	44.9(419/933)	
Preterm birth	6.5(16/245)	6.6(62/933)	.949
Neonatal birthweight	3401.67±498.97	3468.91±561.27	.088
Low birthweight	4.9(12/245)	4.8(45/933)	.961
Small for gestational age	12.2(30/245)	10.5(98/933)	.436
Macrosomia	13.9(34/245)	16.1(150/933)	.399
Large for gestational age	13.5(33/245)	18.5(173/933)	.063
Congenital malformations rate	0.4(1/245)	0.6(6/933)	.670

Data are presented as mean ± SD for continuous variable and % (n/N) for categorical variable. Student t test was used for continuous variables, and the Pearsonχ^2^ test was used for categorical variables with Fisher’s exact test when necessary.

Regarding the main outcome measures, to adjust for the influence of confounding factors, we conducted a multiple logistic regression analysis. The included factors were maternal age, body mass index, duration of infertility, type of infertility (primary/secondary infertility), infertility diagnosis (tubal/male/others), basal antral follicle count, fertilization method (IVF/ICSI) and developmental stage of blastocysts (D5/D6). The unadjusted OR and adjusted OR value with their 95% CIs are presented in **Table 4**. After adjustments for confounding factors, the LBR was higher in the L-FET group (aOR 1.30, 95% CI 1.06-1.58). The specific multiple logistic regression data on LBW are presented in **Supplemental Table 1**. The rate of miscarriage was lower in the L-FET group (aOR 0.63, 95% CI 0.44-0.90). Furthermore, the rates of preterm birth, LBW, SGA, macrosomia and LGA remained consistent with the unadjusted rates, and the rates were comparable between the two groups.

**Table 4:** Unadjusted and adjusted odds ratios of reproductive outcomes of blastocysts transfer following L-FET versus HRT cycles.

	Unadjusted OR (95%CI )	Adjusted OR (95%CI )
Live birth rate	1.37(1.13-1.67)	1.30(1.06-1.58)
Abortion rate	0.60 (0.42-0.86)	0.63(0.44-0.90)
Preterm birth	0.98 (0.56-1.73)	0.99(0.56-1.77)
Low birthweight	0.98(0.51-1.89)	0.97(0.50-1.87)
Small for gestational age	0.84(0.54-1.30)	0.84(0.54-1.29)
Macrosomia	1.19(0.80-1.78)	1.18(0.79-1.78)
Large for gestational age	1.46(0.97-2.19)	1.45(0.97-2.17)

Analysis were adjusted for maternal age, body mass index, duration of infertility , type of infertility(Primary/ Secondary infertility), infertility diagnosis(Tubal/ Male/ Others) , basal antral follicle count, fertilization method(IVF/ICSI) and development stage of blastocysts(D5/D6) .CI, confidence interval.

## Discussion

In summary, the results of this study demonstrate that the L-FET group has a higher LBR and lower miscarriage rate than the HRT group of patients with abnormal ovulation with a single blastocyst transfer. However, the offspring outcomes, including the rates of preterm birth, normal neonatal birthweight, LBW, SGA, macrosomia and LGA, were comparable between the two groups.

### Comparisons With Other Reports

Regarding clinical outcomes, to the best of our knowledge, few studies have explored L-FET and HRT for patients with abnormal ovulation, including polycystic ovary syndrome (PCOS). The first study was published in 2014 and included only 116 PCOS cycles (20). The results of the study showed that compared with those of the HRT group, the clinical pregnancy rate and ongoing pregnancy rate were significantly higher in the L-FET group. In the same year, Li et al. (21) evaluated the clinical efficacy of L-FET on ovulation induction and HRT during endometrial preparation in patients with ovulation disorders. This study suggested that the LBR of patients in the L-FET group (44.6%) was significantly higher than that of patients in the HRT group (32.5%, P < 0.05), while the miscarriage rate (12.0%) was significantly lower than that in the HRT group (21.0%, P < 0.05). A recent study published in 2019. Zhang et al. (9) further explored the clinical outcomes of L-FET and HRT among women with PCOS, and the results suggested that letrozole use for endometrial preparation was associated with higher LBR than use of HRT.

However, one randomized controlled trial of 177 infertile PCOS patients had a different result. After analysis, there were no significant between-group differences in the implantation rate; the chemical, ectopic, and clinical pregnancy rates; the miscarriage rate; or the ongoing pregnancy rate (17). To the best of our knowledge, only one study has explored both clinical outcomes and offspring outcomes. The study was a large retrospective cohort study from Japan published in 2017 (with 110 772 FET cycles included) (18). Compared to those in the HRT group, the clinical pregnancy rate, clinical pregnancy with fetal heartbeat rate, and LBR were significantly higher in the L-FET group, while the miscarriage rate was significantly lower. Neonatal outcomes with the different regimens were mostly similar, which is consistent with our findings. However, the study did not strictly limit the inclusion of patients. According to the description in the article, in Japan, letrozole is used mainly for unexplained infertility, not for people with ovulation disorders. The important limitations of the study are the lack of information concerning the reasons for selecting the specific FET method, parity, the number of previous ART failures, embryo quality and the dose and duration of letrozole intake.

Moreover, the study did not limit the type of ET, including cleavage-stage or blastocyst-stage ET, or the number of transferred embryos. According to current research, there may be differences in clinical and neonatal outcomes between cleavage-stage and blastocyst-stage embryo transfers (22–24). Moreover, the effect of the number of transferred embryos on pregnancy outcome is relatively clear (25, 26). Therefore, to exclude the confounding effects of embryo developmental stage and number of transferred embryos on outcomes, we included only single blastocyst transfer on day 5 or day 6.

### Plausible Biological Mechanisms

There are two possible mechanisms underlying the significant increase in the LBR and decrease in the miscarriage rate. One is that letrozole is a specific third-generation aromatase inhibitor; when the aromatase is inhibited, the conversion of androgens to estrogens decreases, and the decrease in peripheral estrogen leads to increased expression of estrogen receptors and, thus, increased sensitivity to subsequently high estrogen levels, resulting in faster endometrial proliferation and increased blood flow in the uterus and endometrium, with positive effects on implantation (18, 27, 28). Our study also shows that the thickness of the endometrium in the L-FET group was higher than that in the HRT group, which may be related to this mechanism. Another possible mechanism is that letrozole might increase endometrial receptivity by increasing the expression of uterine receptivity such as integrin, L-selectin, leukemia inhibitory factor and pinopods during the implantation window (29), which is beneficial for improving the success rate of frozen embryo transfer.

### Strengths and Limitations

The strengths of this study are twofold. First, we included single-center cycles with full follow-up, cycles performed during the same period, and first transfer cycles, and this study included only the single blastocyst transfer cycle, to control the number of embryos transferred for the assessment of the pregnancy outcomes, as the consequent reduction in twin pregnancies had an impact on neonatal outcomes, which minimized potential bias. Second, our study explored not only the clinical outcomes of the two regimens but also their safety for the offspring, which has always been the focus of research and has always been of profound importance. Our study also has several limitations. First, it is limited by its retrospective nature, and thus, a further prospective study is needed. Second, we did not explore the relevant biological mechanism.

In conclusion, for patients with abnormal ovulation, the L-FET regimen has a higher LBR and lower miscarriage rate than HRT. The neonatal outcomes, including the rates of preterm birth, normal neonatal birthweight, LBW, SGA, macrosomia and LGA, were similar between the two groups. Therefore, these findings imply that letrozole might be a better regimen for FET among women with abnormal ovulation, but further randomized controlled studies with large samples are needed.

## Data Availability Statement

The raw data supporting the conclusions of this article will be made available by the authors, without undue reservation.

## Ethics Statement

The studies involving human participants were reviewed and approved by 2020 Medical Ethics Review No. 126. Written informed consent for participation was not required for this study in accordance with the national legislation and the institutional requirements.

## Author Contributions

JZ and DM designed the study and selected the population to be included and excluded. JZ and ZL were involved in the data extraction and analysis. LS and YG reviewed the data. JZ was involved in drafting this article. All authors contributed to the article and approved the submitted version.

## Conflict of Interest

The authors declare that the research was conducted in the absence of any commercial or financial relationships that could be construed as a potential conflict of interest.

## References

[1] Pereira N Rosenwaks Z. A fresh(er) perspective on frozen embryo transfers. Fertil Steril (2016) 106:257–8. 10.1016/j.fertnstert.2016.06.028 27421613

[2] Gliozheni O Calhaz-Jorge C De Geyter C Kupka MS de Mouzon J Erb K. Assisted reproductive technology in Europe, 2013: results generated from European registers by ESHRE. Hum Reproduct (2017) 32:1957–73. 10.1093/humrep/dex264 29117383

[3] Rienzi L Gracia C Maggiulli R LaBarbera AR Kaser DJ Ubaldi FM. Oocyte, embryo and blastocyst cryopreservation in ART: systematic review and meta-analysis comparing slow-freezing versus vitrification to produce evidence for the development of global guidance. Hum Reprod Update (2017) 23:139–55. 10.1093/humupd/dmw038 PMC5850862 27827818

[4] Groenewoud ER Cohlen BJ Macklon NS. Programming the endometrium for deferred transfer of cryopreserved embryos: hormone replacement versus modified natural cycles. Fertil Steril (2018) 109:768–74. 10.1016/j.fertnstert.2018.02.135 29778369

[5] De Geyter C Calhaz-Jorge C Kupka MS Wyns C Mocanu E Motrenko T. ART in Europe, 2014: results generated from European registries by ESHRE: The European IVF-monitoring Consortium (EIM) for the European Society of Human Reproduction and Embryology (ESHRE). Hum Reproduct (2018) 33:1586–601. 10.1093/humrep/dey242 30032255

[6] Ghobara T Gelbaya TA Ayeleke RO. Cycle regimens for frozen-thawed embryo transfer. Cochrane Database System Rev (2017) 7:CD003414. 10.1002/14651858.CD003414.pub3 28675921 PMC6483463

[7] Glujovsky D Pesce R Sueldo C Quinteiro Retamar AM Hart RJ Ciapponi A. Endometrial preparation for women undergoing embryo transfer with frozen embryos or embryos derived from donor oocytes. Cochrane Database System Rev (2020) 10:CD006359. 10.1002/14651858.CD006359.pub3 33112418 PMC8094620

[8] Mackens S Santos-Ribeiro S van de Vijver A Racca A Van Landuyt L Tournaye H. Frozen embryo transfer: a review on the optimal endometrial preparation and timing. Hum Reproduct (2017) 32:2234–42. 10.1093/humrep/dex285 29025055

[9] Zhang J Liu H Wang Y Mao X Kuang Y. Letrozole use during frozen embryo transfer cycles in women with polycystic ovary syndrome. Fertil Steril (2019) 112:371–7. 10.1016/j.fertnstert.2019.04.014 31126712

[10] Hu YJ Chen YZ Zhu YM Huang HF. Letrozole stimulation in endometrial preparation for cryopreserved-thawed embryo transfer in women with polycystic ovarian syndrome: a pilot study. Clin Endocrinol (2014) 80:283–9. 10.1111/cen.12280 23808904

[11] Samsami A Ghasmpour L Davoodi S Alamdarloo SM Homayoon H. Frozen embryo transfer: Endometrial preparation by letrozole versus hormone replacement cycle: A randomized clinical trial. Int J Reprod BioMed (2019) 17(12):915–22. 10.18502/ijrm.v17i12.5793 PMC6943798 31970313

[12] Franik S Kremer JA Nelen WL Farquhar C. Aromatase inhibitors for subfertile women with polycystic ovary syndrome. Cochrane Database System Rev (2014) 2:Cd010287. 10.1002/14651858.CD010287.pub2 24563180

[13] Franik S Eltrop SM Kremer JA Kiesel L Farquhar C. Aromatase inhibitors (letrozole) for subfertile women with polycystic ovary syndrome. Cochrane Database System Rev (2018) 5:Cd010287. 10.1002/14651858.CD010287.pub3 29797697 PMC6494577

[14] Brown J Farquhar C. Clomiphene and other antioestrogens for ovulation induction in polycystic ovarian syndrome. Cochrane Database System Rev (2016) 12:CD002249. 10.1002/14651858.CD002249.pub5 27976369 PMC6464012

[15] Tulandi T Martin J Al-Fadhli R Kabli N Forman R Hitkari J. Congenital malformations among 911 newborns conceived after infertility treatment with letrozole or clomiphene citrate. Fertil Steril (2006) 85(6):1761–5. 10.1097/01.ogx.0000248776.88202.f7 16650422

[16] Elizur SE Tulandi T. Drugs in infertility and fetal safety. Fertil Steril (2008) 89:1595–602. 10.1016/j.fertnstert.2008.02.092 18519067

[17] Hosseini-Najarkolaei A Moini A. The effect of letrozole versus artificial hormonal endometrial preparation on pregnancy outcome after frozen-thawed embryos transfer cycles: a randomized clinical trial. Reprod Biol Endocrinol (2020) 18(1):115. 10.1186/s12958-020-00675-z 33218350 PMC7678072

[18] Tatsumi T Jwa SC Kuwahara A Irahara M Kubota T Saito H. Pregnancy and neonatal outcomes following letrozole use in frozen-thawed single embryo transfer cycles. Hum Reproduct (2017) 32:1244–8. 10.1093/humrep/dex066 28398491

[19] Dai L Deng C Li Y Zhu J Mu Y Deng Y. Birth weight reference percentiles for Chinese. PloS One (2014) 9:e104779. 10.1371/journal.pone.0104779 25127131 PMC4134219

[20] Hu YJ Chen YZ Zhu YM Huang HF. Letrozole stimulation in endometrial preparation for cryopreserved-thawed embryo transfer in women with polycystic ovarian syndrome: a pilot study. Clin Endocrinol (2014) 80:283–9. 10.1111/cen.12280 23808904

[21] Li SJ Zhang YJ Chai XS Nie MF Zhou YY Chen JL. Letrozole ovulation induction: an effective option in endometrial preparation for frozen-thawed embryo transfer. Arch Gynecol Obstet (2014) 289:687–93. 10.1007/s00404-013-3044-0 24121690

[22] Wang X Du M Guan Y Wang B Zhang J Liu Z. Comparative neonatal outcomes in singleton births from blastocyst transfers or cleavage-stage embryo transfers: a systematic review and meta-analysis. Reprod Biol Endocrinol RB&E (2017) 15:36. 10.1186/s12958-017-0255-4 28472983 PMC5418763

[23] Glujovsky D Farquhar C Quinteiro Retamar AM Alvarez Sedo CR Blake D. Cleavage stage versus blastocyst stage embryo transfer in assisted reproductive technology. Cochrane Database System Rev (2016) 6:Cd002118. 10.1002/14651858.CD002118.pub5 27357126

[24] Kontopoulos G Simopoulou M Zervomanolakis I Prokopakis T Dimitropoulos K Dedoulis E. Cleavage Stage versus Blastocyst Stage Embryo Transfer in Oocyte Donation Cycles. Medicina (Kaunas Lithuania) (2019) 55(6):293. 10.3390/medicina55060293 PMC6631639 31226849

[25] Bhandari S Ganguly I Agarwal P Munaganuru N Gupta N Singh A. Relationship of Number of Embryos Transferred with Perinatal Outcome of Singleton Pregnancy. J Reprod Infertil (2017) 18:179–84. PMC5359855 28377897

[26] Balen AH MacDougall J Tan SL. The influence of the number of embryos transferred in 1060 in-vitro fertilization pregnancies on miscarriage rates and pregnancy outcome. Hum Reproduct (1993) 8:1324–8. 10.1093/oxfordjournals.humrep.a138250 8408536

[27] Casper RF Mitwally MF. Use of the aromatase inhibitor letrozole for ovulation induction in women with polycystic ovarian syndrome. Clin Obstet Gynecol (2011) 54:685–95. 10.1097/GRF.0b013e3182353d0f 22031258

[28] Garcia-Velasco JA. The use of aromatase inhibitors in in vitro fertilization. Fertil Steril (2012) 98:1356–8. 10.1016/j.fertnstert.2012.09.042 23062732

[29] Ganesh A Chauhan N Das S Chakravarty B Chaudhury K. Endometrial receptivity markers in infertile women stimulated with letrozole compared with clomiphene citrate and natural cycles. Syst Biol Reprod Med (2014) 60:105–11. 10.3109/19396368.2013.862316 24304327

