# Supplementary material for: Comparison of Pregnancy and Neonatal Outcomes of Single Frozen Blastocyst Transfer Between Letrozole-Induction and HRT Cycles in Patients With Abnormal Ovulation
Source: Front Endocrinol (Lausanne). 2021 Apr 16;12:664072. doi: 10.3389/fendo.2021.664072 (PMC8087245; doi:10.3389/fendo.2021.664072)
Supplement: Supplementary file 1 [file DataSheet_1.docx]

Supplementary Table 1 Multivariable logistic regression analysis to account for confounding variables of LBR

|  | Adjusted OR  (95%CI ) | *P* value |
| --- | --- | --- |
| Maternal age(y) | 0.94(0.92-0.96) | <.001 |
| Body mass index(kg/m2) | 0.99(0.97-1.02) | .565 |
| Duration of Infertility (y) | 0.99(0.97-1.03) | .899 |
| Type of infertility(Primary/ Secondary infertility) | 0.99(0.83-1.19) | .979 |
| Infertility diagnosis(Tubal/ Male/ Others) | 0.98(0.89-1.07) | .640 |
| Basal antral follicle count | 1.01(0.99-1.02) | .110 |
| Fertilization method(IVF/ICSI) | 1.50(1.25-1.80) | <.001 |
| Development stage of blastocysts(D5/D6) | 0.64(0.54-0.75) | <.001 |
| Endometrial preparation protocols(L-FET/ HRT) | 1.30(1.06-1.58) | .010 |
| Note: Variable entered in the logistics regression model listed. CI=confidence interval | | |
